# Prostaglandin E_1_ in ischemic retinal diseases: mechanisms, evidence, and clinical perspectives

**DOI:** 10.3389/fmed.2026.1749690

**Published:** 2026-02-09

**Authors:** Dario Rusciano, Caterina Gagliano, Alessandro Avitabile, José Fernando Maya-Vetencourt

**Affiliations:** 1 Neurovisual Science Technology (NEST), Catania, Italy; 2 Faculty of Medicine and Surgery, University of Enna “Kore”, Enna, Italy; 3 Mediterranean Foundation “G.B. Morgagni”, Catania, Italy; 4 Neurovisual Science Technology (NEST), Catania, Italy; 5 Department of Biology, Physiology Institute, University of Pisa, Pisa, Italy

**Keywords:** anti-inflammatory activity, anti-thrombotic and rheological effects, diabetic retinopathy, endothelial protection, neuroprotective and mitochondrial benefits, non-arteritic ischemic optic neuropathy, retinal vein and artery occlusions, vasodilation

## Abstract

Ischemic retinal diseases, including retinal vein and artery occlusions, diabetic retinopathy, and non-arteritic ischemic optic neuropathy, are major causes of vision loss worldwide and remain incompletely addressed by current therapies. Prostaglandin E1 (PGE_1_) has emerged as a promising therapeutic candidate due to its unique pharmacological profile, encompassing vasodilation, anti-thrombotic and rheological effects, endothelial protection, anti-inflammatory activity, and potential neuroprotective and mitochondrial benefits. Preclinical studies demonstrate that PGE_1_ improves retinal perfusion, reduces oxidative stress and edema, and promotes neuronal survival, while early clinical experiences, though limited in size, suggest favorable effects on visual outcomes and microcirculation, with an acceptable safety profile. However, evidence is limited by small sample sizes, heterogeneity, and delivery challenges. Emerging approaches, including sustained-release formulations, intravitreal delivery, and combination therapies, along with imaging biomarkers for patient selection, offer avenues to optimize clinical translation. While early clinical experiences—particularly in acutely treated central retinal artery occlusion—suggest potential benefits on retinal perfusion and visual outcomes, the current evidence remains limited by small, heterogeneous, and predominantly non-randomized studies. Consequently, PGE₁ should be regarded as an investigational or adjunctive approach, warranting further evaluation in well-designed, controlled clinical trials before any routine clinical adoption.

## 1 Introduction

Ischemic retinal diseases—including retinal vein occlusion [branch (BRVO) and central (CRVO)], central and branch retinal artery occlusion (CRAO/BRAO), diabetic retinopathy (DR), and non-arteritic ischemic optic neuropathy (NAION)—represent major global causes of visual impairment and blindness. These disorders result from reduced or interrupted retinal blood flow, leading to hypoxia, capillary non-perfusion, edema, and secondary neuronal injury. Visual loss due to ischemia is often severe and irreversible, particularly when treatment is delayed. Epidemiological analyses indicate that diabetic retinopathy affects more than 100 million individuals worldwide and remains the leading cause of blindness in working-age adults (1); CRVO and CRAO together account for several thousand new vision-threatening cases each year, frequently with poor prognoses despite prompt intervention (2).

Current management strategies differ according to disease entity but share fundamental limitations. In retinal vein occlusion and diabetic macular edema, intravitreal anti-VEGF agents and corticosteroids effectively reduce macular edema and neovascular proliferation but do not address the underlying ischemia or neuronal injury (3). Recent advances in biomimetic and nanocarrier-based delivery systems aim to overcome these shortcomings by enhancing drug penetration, prolonging intraocular retention, and reducing injection frequency (4). In CRAO and BRAO, treatment options remain limited and largely unsatisfactory, with no therapy reliably restoring vision (5). Likewise, for non-arteritic ischemic optic neuropathy, no consistently effective treatment exists (6). Across these disorders, persistent capillary non-perfusion, oxidative stress, inflammation, and ganglion cell death remain incompletely addressed by current therapies.

Prostaglandin E₁ (PGE₁, alprostadil) has emerged as a potential therapeutic candidate because of its multifaceted pharmacological profile, encompassing vasodilatory, anti-thrombotic, rheological, endothelial-protective, and anti-inflammatory effects, together with possible neuroprotective and mitochondrial benefits (7). Beyond ophthalmology, PGE1 improves endothelial progenitor cell function, reduces oxidative and inflammatory damage, and enhances tissue perfusion in peripheral and cerebral ischemic models (7).

Recent preclinical and early clinical data suggest that these effects may extend to the retina. Several studies have reported that intravenous or liposomal PGE₁ improves retinal perfusion and visual outcomes in CRAO. In a prospective series, lipo-PGE₁ administration in acute CRAO significantly improved best-corrected visual acuity (BCVA) and retinal thickness at 1 and 3 months without serious adverse events (8). A recent systematic review confirmed these findings, highlighting consistent visual and perfusion improvements with an acceptable safety profile (9). A retrospective comparative study found that patients receiving early intravenous PGE₁ achieved greater visual recovery than those receiving standard therapy alone (10). Moreover, angiographic evidence of reperfusion after PGE₁ infusion has been documented, accompanied by recovery of counting-finger vision in previously amaurotic eyes (11). Collectively, these findings indicate that PGE_1_ may exert beneficial effects on both the vascular and neuronal components of retinal ischemia.

Nevertheless, important gaps remain. Most available studies are limited by small sample sizes, heterogeneous dosing schedules, differences in route of administration (intravenous, liposomal, or potential intraocular delivery), and variability in timing relative to symptom onset. Furthermore, imaging biomarkers capable of identifying patients most likely to benefit from PGE₁ remain to be standardized. Larger, randomized, controlled trials are needed to confirm efficacy and to define optimal protocols for clinical use (8–10).

The aim of this review is therefore to synthesize current knowledge on PGE₁ in ischemic retinal diseases—examining its mechanisms of action, preclinical and clinical evidence, delivery strategies, and safety profile—and to outline future perspectives, including combination therapies, sustained-release formulations, and biomarker-driven patient selection to enhance translation from bench to bedside. Although some of the available evidence is dated, this largely reflects the scarcity of recent ophthalmic research on PGE₁ rather than the obsolescence of the earlier findings, highlighting a clear need for renewed scientific and clinical investigation in this field. Importantly, PGE₁ is not included in current treatment guidelines for ischemic retinal diseases, including CRAO, RVO, diabetic retinopathy, or non-arteritic ischemic optic neuropathy. At present, its use remains off-label and largely confined to exploratory clinical settings. Accordingly, this review does not advocate routine clinical use, but rather aims to critically examine existing evidence, delineate its limitations, and identify the most rational indications and trial designs for future investigation.

## 2 Pathophysiology of ischemic retinal injury

The retina is one of the most metabolically demanding tissues in the body, with a high oxygen consumption per unit volume and negligible capacity for anaerobic energy production. This metabolic demand confers intrinsic vulnerability to even brief interruptions in perfusion (12).

### 2.1 Retinal microcirculation and vulnerability to ischemia

The retinal vascular architecture is organized into two principal systems: the inner retinal circulation, derived from the central retinal artery and serving the inner retinal layers (ganglion, inner plexiform and nuclear layers), and the **outer retina**, which is avascular and relies on diffusion from the choroidal circulation and the retinal pigment epithelium. Because the outer retina is not directly vascularized, it is especially susceptible to hypoxia when the choroidal supply is compromised or when inner retinal swelling impinges on diffusion (13).

Within the inner retina, microcirculatory units comprise arterioles, capillaries, and venules wrapped by pericytes and ensheathed by Müller glial processes. The narrow lumen of capillaries, tight coupling with metabolic demand, and limited collateralization mean that even modest vascular insults can produce localized ischemia. Studies of human donor retinal microcirculation reveal heterogeneity in capillary density and flow reserve, especially in the perifoveal and parafoveal zones, which may contribute to the spatial predilection of ischemic damage in diseases such as central retinal vein occlusion or diabetic retinopathy (14).

Ischemic insult to the retinal microcirculation can result from either vascular occlusion (arterial or venous) or from endothelial dysfunction driven by systemic risk factors (diabetes, hypertension, atherosclerosis). In the case of venous occlusion, elevated venous pressure leads to capillary stasis, hemorrhage, and nonperfusion, while arterial occlusion sharply halts blood supply to downstream capillaries.

### 2.2 Key mechanisms: hypoxia, endothelial dysfunction, inflammation, neuronal loss

Once retinal perfusion is compromised, it initiates a deleterious and self-perpetuating cascade of pathological events. A primary and immediate consequence is tissue hypoxia, which stabilizes hypoxia-inducible factors (HIFs), particularly HIF-1α, that function as master transcriptional regulators of oxygen homeostasis (15). In an initially adaptive effort to salvage compromised tissue, these factors upregulate a suite of genes encoding angiogenic mediators such as Vascular Endothelial Growth Factor (VEGF) and Angiopoietin-2 (ANGPT2), alongside glycolytic enzymes and erythropoietin, thereby promoting vascular perfusion and metabolic reprogramming (15, 16). However, when HIF activation becomes persistent within the retinal environment, this response becomes maladaptive, driving pathological angiogenesis, increasing vascular permeability, and ultimately exacerbating macular edema and ischemic injury (17–19).

Concurrently, the reduced supply of oxygen and nutrients impairs mitochondrial oxidative phosphorylation, leading to a marked generation of reactive oxygen species (ROS) and direct mitochondrial damage. This state of profound oxidative stress inflicts injury upon both the delicate vascular endothelial cells and the neuronal elements of the retina. Over time, ROS perpetuate a destructive cycle by further promoting endothelial dysfunction, causing mitochondrial DNA damage, and ultimately triggering cell death, establishing oxidative stress as a recognized central pathway underlying both microvascular degeneration and neuronal loss in ischemic retinal diseases (20). The resulting endothelial dysfunction critically leads to a loss of tight junction integrity, particularly within the inner blood-retina barrier (BRB). This breach in the retinal vasculature permits the infiltration of plasma proteins and immune cells, significantly exacerbating edema and initiating a robust inflammatory response (21). This process is often amplified in the context of ischemia–reperfusion injury, which further intensifies the inflammatory cascade through mechanisms such as increased leukocyte adhesion mediated by ICAM-1 and selectins, the activation of resident microglia, and the release of pro-inflammatory cytokines including IL-1β, TNF-α, and IL-6. These inflammatory events engage in a vicious cycle with ongoing oxidative stress, collectively contributing to the damage of retinal cells (21).

The culmination of this pathophysiological cascade is the irreversible loss of retinal neurons and the activation of glial cells. Retinal ganglion cells (RGCs), along with other inner retinal neurons like amacrine and bipolar cells, exhibit a high degree of vulnerability to both metabolic stress and excitotoxic injury (22). Prolonged ischemia triggers the excessive release of the neurotransmitter glutamate, leading to calcium overload within neurons and the activation of both apoptotic and inflammatory intracellular cascades (22). This sequence of events is consistently reflected in experimental ischemia–reperfusion models, which demonstrate characteristic findings such as inner retinal thinning, a significant loss of RGCs, a decreased b-wave amplitude on electroretinography indicative of inner retinal dysfunction, and reactive Müller gliosis (23, 24).

### 2.3 Where standard therapies fall short

Therapeutic strategies in ischemic retinal diseases have evolved to target downstream consequences rather than upstream ischemia. In retinal vein occlusion and diabetic macular edema, intravitreal anti-VEGF agents and corticosteroids are highly effective at reducing vascular permeability and neovascular proliferation. However, these treatments do not directly restore microcirculation, address nonperfusion, or prevent neuronal degeneration in ischemic zones. In fact, anti-VEGF therapies may worsen retinal ischemia or fail to reduce nonperfusion areas (25–27). They often require repeated administration and may not penetrate ischemic or occluded capillaries.

Moreover, in retinal and optic nerve arterial occlusions, therapies aimed at reperfusion (e.g., thrombolysis, hyperbaric oxygen) have shown variable efficacy, mostly limited by narrow time windows and risk of reperfusion injury. Many patients present too late for these interventions. Even when perfusion is restored, microvascular rarefaction, endothelial injury, and capillary dropout may prevent true functional recovery (as evidenced by worsening peripheral ischemia after treatment) (28).

Imaging biomarkers—such as OCT-A quantification of capillary density, non-perfusion maps, or perfusion indices—are increasingly used to stratify disease severity and monitor response (e.g., deep capillary nonperfusion predicting DR progression) (29). However, standardization of these imaging markers across devices and clinical trials is lacking, and predictive thresholds for therapeutic benefit remain undefined (30, 31).

Within this pathophysiological framework, the need to explore novel mechanistic insights and time-sensitive therapeutic strategies for ischemic retinal injury becomes apparent, rather than defining a specific therapeutic window for PGE₁ at this stage. Because PGE₁‘s mechanisms include vasodilation, endothelial protection, antithrombotic effects, anti-inflammatory modulation, and potentially mitochondrial support, it is ideally placed to act upstream of irreversible tissue injury. For maximal efficacy, PGE₁ would need to be delivered early, before capillary dropout and neuronal apoptosis become irreversible, and within a timeframe in which reperfusion remains meaningful. Indeed, a retrospective cohort of CRAO patients receiving PGE₁ within 24 h showed superior visual recovery compared to conventional therapy (10). The urgency is emphasized by estimates that retinal ganglion cells may suffer irreversible injury in as little as 12–15 min after occlusion (32).

## 3 Prostaglandin E₁: biological mechanisms and clinical context

### 3.1 Biological functions of prostaglandin E₁

PGE₁ is a naturally occurring lipid mediator derived from the cyclooxygenase (COX) pathway that acts primarily through prostanoid (EP) receptors. Although it shares certain pharmacological properties with other prostaglandins, PGE₁ is distinct from PGE₂ in its receptor affinity and downstream signaling, leading to characteristic biological actions such as vasodilation, inhibition of platelet aggregation, and modulation of vascular smooth muscle tone. Increasing attention has recently been directed toward prostanoid signaling within the retina, where different prostaglandin families and receptor subtypes contribute to the regulation of vascular homeostasis, inflammation, and neuronal survival. As highlighted by (33), these mechanisms are relevant to a range of ocular disorders, including diabetic retinopathy, age-related macular degeneration (AMD), retinal vascular occlusions, and uveitis (Table 1).

**Table 1:** Summary of key preclinical and clinical studies investigating PGE₁ in ischemic and vascular disorders relevant to the retina.

Study/Year	Model / population	Route & dose (reported)	Key findings / outcomes	Main limitations / gaps
Takai et al. 2013 (34)	Acute central retinal artery occlusion (CRAO); retrospective series (10 eyes, 9 pts)	Systemic: IV 40 μg twice daily ×5 days, then oral 30 μg TID ≥ 1 month	Rapid restoration of retinal blood flow; improved best-corrected visual acuity in several eyes; well tolerated	Small, non-randomized, retrospective; no sham/control group; possible selection bias
Suzuki et al. 2022 (8)	Acute CRAO (*n* = 21) — prospective/observational (lipo-PGE₁)	Intravenous liposomal PGE₁ 10 μg/day for 7–14 days (protocols varied)	Anatomical and functional improvement reported (FA reperfusion, OCT changes, BCVA gains) in treated patients	Non-randomized, relatively small; heterogeneity in timing and adjunctive care; single-center
Serhan et al. 2025 (9) (systematic review)	Systematic review of IV PGE₁ for CRAO/BRAO	Aggregated clinical data (various IV regimens)	Synthesizes case series and observational data suggesting potential benefit when given early; calls for RCTs	Heterogeneous, low-quality primary studies; no definitive RCT evidence
Ling et al. 2016 (39)	Rat permanent MCAO (ischemic stroke model) — preclinical	lipo-PGE₁ 10 μg/kg/day IP (or IV equiv.) × 6 days (started 24 h post-MCAO)	Increased peri-infarct vascular density, enhanced neurogenesis, improved neurological recovery — supports angiogenesis/neurogenesis	Animal model (brain ischemia), not retina; dose translation to humans uncertain
Shen et al. 2021 (38)	In vitro mouse cortical neurons (hemin toxicity model)	PGE₁ (concentrations in cell assays)	Activates Nrf2/HO-1 pathway, reduces oxidative stress and neuronal apoptosis — plausible antioxidant / cytoprotective mechanism	In vitro neuronal model (not retinal in vivo); concentration/dose not directly translatable to humans
Li et al. 2013 (36)	Acute lower-limb ischemia [Prospective clinical cohort (204 patients)]	IV lipo-PGE₁ 20 μg/day ×12–14 days (post-surgery)	Improved limb salvage and outcomes as adjuvant to surgery; supports vascular, rheological, anti-inflammatory properties of lipo-PGE₁	Peripheral vascular disease (not ocular); extrapolation to retina indirect

The table focuses on mechanistic and biological effects of PGE₁ relevant to retinal ischemia. The table highlights, PGE_1_ vasodilatory, endothelial-protective, and neuroprotective effects, as well as current limitations related to small sample sizes, heterogeneous protocols, and the lack of randomized clinical trials.

### 3.2 Clinical applications of prostaglandin E₁

Recent investigations have explored the potential of PGE₁ to counteract ischemic injury in the retina by enhancing microcirculation and promoting cellular resilience. In a multicenter retrospective study of central retinal artery occlusion (CRAO), early administration of intravenous PGE₁—initiated within 24 h of onset and followed by oral continuation—was associated with improved visual outcomes compared with standard care, suggesting that timely vasodilation and reperfusion of the ischemic retina may be achievable (10). These findings are consistent with earlier reports by (34), who documented rapid restoration of retinal blood flow and improvement in visual acuity after short-term intravenous PGE₁ therapy in CRAO patients. More recently (8) confirmed anatomical and functional recovery following intravenous liposomal PGE₁, further supporting the feasibility of systemic administration in acute retinal ischemia.

Earlier clinical evidence in patients with peripheral vascular disease and diabetes similarly demonstrated that intravenous PGE₁ significantly increases both systolic and diastolic flow velocities in the ophthalmic and central retinal arteries, indicating enhanced ocular perfusion and microcirculatory support (35). Comparable benefits have also been observed in peripheral ischemic conditions: in a large clinical cohort of patients with acute lower-limb ischemia, (36) reported that intravenous liposomal PGE₁ improved tissue perfusion, reduced inflammatory markers, and enhanced post-surgical outcomes, reinforcing the systemic vasoprotective and rheological potential of this agent.

Beyond its hemodynamic actions, PGE₁ exhibits anti-thrombotic and rheological properties through inhibition of platelet aggregation and modulation of blood flow dynamics. Although contemporary retinal-specific studies are limited, systemic investigations have demonstrated its ability to reduce vascular occlusion risk in other contexts, such as the prevention of veno-occlusive disease (37). These findings are consistent with the notion that PGE₁ promotes vascular patency by improving blood rheology and endothelial stability.

Emerging evidence also points to PGE₁‘s role in endothelial protection and anti-inflammatory regulation. In systemic sclerosis, for example, (7) reported that PGE₁ restored the function of endothelial progenitor cells impaired by disease, suggesting a broader endothelial reparative effect. While most research on prostanoid-mediated inflammation has centered on PGE₂ and COX-2–dependent pathways in retinal glia and vasculature, the known endothelial actions of PGE₁—including vasodilation, anti-aggregatory activity, and improved cell survival—imply a potential to mitigate oxidative and inflammatory stress in retinal microvessels (7, 33).

At the molecular level, PGE₁ has been shown to activate antioxidant signaling through the Nrf2/HO-1 axis, reducing neuronal oxidative stress and apoptosis *in vitro*, as demonstrated by (38). Preclinical studies further support a neuroprotective and potentially mitochondrial-stabilizing role for PGE₁. In animal models of cerebral ischemia, administration of liposomal PGE₁ (lipo-PGE₁) enhanced angiogenesis, promoted neural progenitor cell proliferation, and improved neurological recovery, pointing to reparative and neurogenic effects beyond vascular dilation (39). The clinical observations in CRAO align with these findings, as early treatment was associated with cytoprotective effects and functional visual recovery (10). Although mitochondrial-specific mechanisms have not been fully characterized in retinal tissue, the broader pattern of reduced oxidative injury and inflammation suggests that PGE₁ may sustain neuronal and glial viability under ischemic stress.

### 3.3 Current challenges and limitations

Despite its therapeutic promise, PGE₁‘s pharmacological application is limited by its short systemic half-life and metabolic instability. Classic pharmacokinetic studies in animal models have shown that its primary metabolite, 13,14-dihydro-15-keto-PGE₁, has a terminal half-life of approximately 25–34 min, which constrains sustained systemic activity (40). Consequently, most clinical protocols rely on continuous intravenous infusion or repeated daily administration. As described by (10) and confirmed by (9), CRAO treatment typically involves intravenous PGE₁—sometimes followed by oral continuation—to maintain therapeutic exposure. However, systemic delivery, while achieving broad tissue distribution, carries potential risks such as hypotension, headache, and bleeding, reflecting PGE₁’s potent vasodilatory and antiplatelet actions.

Experimental data indicate that effective doses in animal studies (for example, 10 μg/kg/day of lipo-PGE₁ in ischemic stroke models) may not be directly translatable to safe, practical regimens in humans, as such doses could lead to systemic adverse effects if repeated chronically (39). Furthermore, ocular delivery poses additional barriers: the blood–retinal barrier, rapid systemic metabolism, and dilution effects all limit retinal bioavailability. To date, intravitreal or periocular administration of PGE₁ has not been systematically evaluated in modern trials. Novel delivery platforms, including liposomal or sustained-release formulations, may help to overcome these obstacles by prolonging exposure and minimizing systemic side effects.

In terms of safety, no major adverse events were reported in the recent CRAO study using early intravenous PGE₁ (10), yet comprehensive safety assessment requires larger, controlled investigations. The efficacy of PGE₁ also appears to be highly time-dependent—benefits are most evident when administered soon after ischemic onset—underscoring the importance of early diagnosis and appropriate patient selection. Ultimately, while current human evidence remains limited to small case series and retrospective analyses, the convergence of hemodynamic, endothelial, and neuroprotective effects provides a compelling rationale for further randomized clinical trials. Optimizing delivery strategies and establishing validated biomarkers of response will be essential to determine PGE₁‘s definitive place in the management of ischemic and neovascular retinal disorders.

Collectively, these biological and pharmacological properties provide a coherent mechanistic rationale for considering PGE₁ in ischemic retinal conditions. However, mechanistic plausibility alone is insufficient to justify clinical translation. The relevance of these effects must therefore be evaluated within controlled experimental systems and, ultimately, in human disease contexts characterized by distinct temporal, vascular, and cellular constraints.

## 4 Evidence from preclinical research

The transition from theoretical mechanism to therapeutic application hinges on robust preclinical evidence, which serves to delineate the pathological timeline of disease and validate a candidate drug’s efficacy in controlled experimental settings. For PGE₁ establishing its potential in ischemic retinal diseases requires a meticulous examination of data derived from models of retinal and optic nerve ischemia, as well as insights from its application in other ischemic organs that share pathophysiological parallels with the retina. This section synthesizes the existing preclinical foundation for PGE₁, evaluating the direct, albeit limited, evidence from ocular models, the compelling translational data from cerebral and cardiac studies, and the critical limitations that currently constrain a full understanding of its therapeutic profile for ophthalmic use.

### 4.1 Experimental models of retinal and optic nerve ischemia

Recent work in rodent models has clarified the timeline and nature of damage following retinal ischemia/reperfusion (I/R). In Brown-Norway rats subjected to elevated intraocular pressure (IOP) for 60 min followed by reperfusion, optic nerves show early microglial activation (as soon as 12 h), followed by neurofilament degeneration and demyelination by days 3–7 (41). Similarly, rat studies with elevated IOP (140 mmHg for 1 h) demonstrated not only retinal morphological damage, but also optic nerve cell infiltration, microglial activation, and structural degeneration over 21 days (42). These studies establish that both retinal and optic nerve neurons are vulnerable early on and that glial/inflammatory responses precede overt neuronal loss.

While the evidence from cerebral and cardiac models is promising, the preclinical data for PGE₁ in the context of retinal and optic nerve ischemia come with significant limitations. A primary constraint is the sheer lack of well-controlled animal studies directly within these ocular tissues; there is very little specific data on how PGE₁ affects neuronal survival, edema, or oxidative stress in the retina, leaving us to rely heavily on human case series and inferences from other organs.

Furthermore, while studies in the brain and heart clearly show that the timing of administration and the dosage are critical, the optimal therapeutic window and formulation for the retina remain entirely unestablished. This challenge is compounded by the heterogeneity of preclinical models themselves, as different methods of inducing ischemia—such as elevated intraocular pressure versus arterial occlusion—can produce distinct pathological cascades, making it difficult to generalize results. Adding another layer of complexity, many studies focus on anatomical or biochemical endpoints, while robust, long-term assessments of functional recovery, like electroretinography (ERG) or optic nerve conduction, are less common. Finally, a comprehensive understanding of the safety profile is often missing, as detailed reporting of adverse effects, particularly for ocular application, is frequently absent from preclinical work.

Beyond retinal ischemia–reperfusion models, experimental paradigms such as optic nerve crush (ONC) are widely used to study axonal injury, retinal ganglion cell degeneration, and secondary ischemic mechanisms affecting the optic nerve. To date, however, no studies have directly evaluated the effects of PGE₁ in ONC or other optic nerve–specific ischemia models. As such, any potential neuroprotective role of PGE₁ in these contexts remains speculative and warrants targeted experimental investigation.

In sum, preclinical evidence suggests that PGE₁ has promising effects on perfusion, reducing oxidative stress, and improving functional outcomes in non-ocular ischemic models. Retinal/optic nerve specific preclinical work remains limited in scope. To validate translatability, more studies are needed that use retina/optic nerve ischemia models, assess neuronal survival and function, optimize dosing and timing, and document safety in ocular tissues.

### 4.2 Translational insights from cerebral and cardiac ischemia

Direct preclinical evidence for PGE₁ in retinal or optic nerve ischemia is limited, with only a retrospective human study in central retinal artery occlusion suggesting potential benefits for visual acuity and edema reduction (10). However, robust mechanistic insights from other ischemic tissues support its translatability. In a rat model of ischemic stroke, delayed treatment with lipo-PGE₁ enhanced angiogenesis and neurogenesis, improving functional recovery (39). Similarly, in a rat model of coronary micro-embolization, PGE₁ pre-treatment improved microvascular function, preserved mitochondrial integrity, and mitigated oxidative stress by protecting antioxidant enzymes (43).

Collectively, these findings from the brain and myocardium—tissues that share a vulnerability to ischemia and reperfusion injury with the retina—suggest PGE₁ can modulate perfusion, reduce oxidative damage, and improve outcomes. While direct evidence for neuronal survival in the visual system remains sparse, these parallel mechanisms indicate a strong therapeutic potential.

## 5 Clinical evidence in ischemic retinal diseases

Ischemic retinal diseases constitute a heterogeneous group of disorders with distinct pathophysiology, natural history, and therapeutic goals. Central retinal artery occlusion (CRAO), retinal vein occlusion (RVO), diabetic retinopathy, and ischemic optic neuropathies differ substantially in terms of reversibility, timing of intervention, and mechanisms of tissue injury. Consequently, the rationale for PGE₁ therapy and the strength of available evidence vary considerably across these conditions and must be interpreted in a disease-specific manner (Table 2).

**Table 2:** Summary of key clinical studies evaluating PGE₁ in ocular ischemic diseases.

Study / year	Indication (*n*)	Treatment protocol	Main outcomes	Safety and tolerability	Key remarks / limitations
Steigerwalt et al. 2008 (44)	Non-arteritic anterior ischemic optic neuropathy (NA-AION); 8 treated + 7 controls	IV PGE₁ 40 μg/day × 5 days + corticosteroids vs. aspirin + steroids	VA improved in 7/8 treated eyes; faster resolution of disc edema	No adverse reactions	Small, non-randomized series; first clinical use demonstrating microcirculatory benefit
Steigerwalt et al. 2014 (45)	Chronic diabetic ischemic eye (case report)	IV PGE₁ 40 μg/day × 5 days → oral 30 μg t.i.d. long-term	Improved and sustained perfusion; reduced non-perfusion area over 4.5 years	Well tolerated	Anecdotal single-case; suggests endothelial recovery with maintenance dosing
Takai et al. 2013 (34)	Central retinal artery occlusion (CRAO); 10 eyes	IV PGE₁ 40 μg b.i.d. × 5 days → oral 30 μg t.i.d. ≥ 1 month	Mean BCVA improved from 2.67 → 0.52 logMAR (*p* = 0.005); arterial +151%, venous +191% dilation; earlier treatment → greater gain	One mild local reaction only	Retrospective; clear correlation between timing and outcome; no control arm
Suzuki et al. 2022 (8)	CRAO; 21 eyes	IV liposomal PGE₁ 10 μg/day × 7–14 days	Mean BCVA 2.18 → 1.54 logMAR (1 mo, *p* < 0.05); reperfusion on FA/OCT; OIR correlated with VA gain	No systemic AEs	Prospective but non-randomized; moderate sample; supports dose feasibility and biomarker correlation
Sano et al. 2025 (10)	CRAO; retrospective cohort	IV PGE₁ initiated ≤ 24 h vs. standard therapy	Superior VA at 1 month in PGE₁ group; baseline MRT negatively correlated with VA gain	No hypotension or bleeding	Confirms time-sensitive benefit; supports early administration
Serhan et al. 2025 (9)	Systematic review of 6 studies (21 patients) with CRAO/BRAO	IV PGE₁ various regimens (5–14 days)	Significant VA gain (Wilcoxon V = 231, p < 0.05); best results ≤ 48 h from onset	No serious AEs reported	Heterogeneous datasets; supports need for RCTs
Steigerwalt et al. 2011 (35)	Non-arteritic posterior ischemic optic neuropathy (NAPION); single case	IV PGE₁ 40 μg/day × 5 days + steroids	VA improved from 4/10 → 11/10; stable at 12 months; resolved VF defects	No AEs	Suggests posterior optic-nerve reperfusion; single-patient observation
Chacko et al. 2023 (11)	CRAO (case report)	Local (intra-arterial) PGE₁ infusion	Documented angiographic reperfusion and VA recovery to CF level	Not reported	Isolated case; first evidence of local delivery; experimental only

This table summarizes experimental and clinical observations and is intended to complement, not replace, the mechanistic overview provided in Table 1. AEs, adverse events; BCVA, best-corrected visual acuity; CF, counting fingers; FA, fluorescein angiography; MRT, maximal retinal thickness; OIR, optical intensity ratio; RCT, randomized controlled trial; VA, visual acuity.

Over the past two decades, incremental clinical experience has progressively outlined a potential therapeutic window for systemic PGE₁ in ocular ischemic diseases. Early reports emerged from compassionate-use observations and were later followed by structured prospective and retrospective analyses. Although the cumulative evidence remains limited in size, these studies collectively suggest that timely administration of PGE₁ can improve retinal perfusion and functional recovery in conditions otherwise resistant to conventional therapy.

The first ophthalmic use of intravenous PGE₁ was documented by Steigerwalt et al. (44) in patients with non-arteritic anterior ischemic optic neuropathy (NA-AION), a disorder lacking effective pharmacological options. In that comparative study, eight eyes received short-term intravenous PGE₁ combined with corticosteroids and were compared with seven controls treated with aspirin and oral steroids. Visual acuity improved in nearly all treated eyes but in less than half of the controls, and optic-disc edema resolved more rapidly in the PGE₁ group. No adverse reactions occurred. The authors attributed these improvements to restoration of optic-nerve head microcirculation, introducing the concept of pharmacological microvascular rescue through systemic vasodilators.

In a subsequent report, the same group described long-term follow-up of an ischemic diabetic eye treated with intravenous and then oral PGE₁, showing progressive recovery of capillary perfusion and durable stabilization over 4.5 years (45). Fluorescein angiography revealed partial reopening of capillary beds and contraction of non-perfused zones. Although anecdotal, this case suggested that repeated or maintenance administration of PGE₁ may support endothelial repair in chronic diabetic microangiopathy.

Among ischemic retinal conditions, CRAO currently represents the most biologically plausible and clinically supported indication for PGE₁ therapy. The abrupt, flow-limiting nature of arterial occlusion, the absence of effective standard treatments, and the strong time-dependence of retinal survival together create a narrow therapeutic window in which pharmacological reperfusion and endothelial protection may yield meaningful benefit.

Clinical interest subsequently focused on central retinal artery occlusion (CRAO), where reperfusion must occur rapidly to preserve vision. In a retrospective series by (34), 10 eyes received intravenous PGE₁ (40 μg twice daily for 5 days) followed by oral continuation. Mean best-corrected visual acuity (BCVA) improved from 2.67 ± 0.54 to 0.52 ± 0.62 logMAR after 1 month (*p* = 0.005). Quantitative fundus analysis showed an immediate increase in vascular calibers—arterial diameters by about 150% and venous by 190%—indicating marked hemodynamic improvement. The degree of visual recovery correlated inversely with time to treatment, confirming that efficacy depends strongly on early intervention.

A prospective observational study by (38) used a liposomal formulation of PGE₁ (10 μg/day i.v. for 7–14 days) in 21 eyes with CRAO. Mean BCVA improved significantly at both one and 3 months (from 2.18 ± 0.60 to 1.54 ± 0.84 and 1.53 ± 0.88 logMAR; *p* < 0.05). Fluorescein angiography demonstrated partial or complete reperfusion in most eyes, and OCT confirmed reduction of inner-retinal edema. Baseline optical-intensity ratios correlated with functional recovery, suggesting a structural biomarker of responsiveness. No systemic adverse events were reported.

Additional confirmation came from (10), who retrospectively compared CRAO patients treated with intravenous PGE₁ within 24 h to those managed with standard care. Visual outcomes at 1 month were significantly better in the PGE₁ group, while greater initial retinal thickening predicted poorer prognosis—again underscoring the importance of rapid administration. No hypotension, headache, or bleeding episodes occurred, confirming the safety of short intravenous regimens.

A systematic review by (9) synthesized six available clinical series (21 patients total) with CRAO or branch RAO treated using intravenous PGE₁. Across studies, visual-acuity gains were statistically significant (Wilcoxon V = 231; *p* < 0.05). Effective outcomes were consistently associated with treatment initiation within 48 h and continuous infusion for 5–7 days. Although the evidence remains heterogeneous and largely non-randomized, the reproducibility of benefit and absence of serious adverse effects lend credibility to these findings.

Evidence supporting the use of PGE₁ in retinal vein occlusion (RVO) or diabetic retinopathy remains speculative. To date, no controlled clinical trials have evaluated PGE₁ in these conditions, and available rationale is largely extrapolated from pathophysiological considerations and non-ocular ischemia models. While venous stasis, endothelial dysfunction, and secondary ischemia are shared features, the chronic and multifactorial nature of these diseases markedly differs from acute arterial occlusion. Accordingly, any application of PGE₁ in RVO or diabetic retinopathy should be regarded as hypothesis-generating rather than evidence-based.

The therapeutic rationale has also been extended to non-arteritic ischemic optic neuropathy (NAION) variants. A case of non-arteritic posterior ischemic optic neuropathy (NAPION) treated with intravenous PGE₁ plus corticosteroids achieved visual improvement from 4/10 to 11/10 Snellen, maintained for 12 months (46). The accompanying resolution of visual-field loss and normalization of optic-disc appearance suggested restoration of posterior ciliary-artery perfusion.

Across all ophthalmic studies, the safety profile of PGE₁ has been consistently favorable. Minor side effects such as transient flushing or local discomfort have been reported only occasionally, with no cases of severe hypotension or bleeding (8, 34). Even in elderly or diabetic patients, intravenous administration for 5–14 days was well tolerated. Broader vascular studies confirm a similar pattern of mild, reversible adverse reactions (10). Evidence for intra-arterial or intra-ocular delivery remains anecdotal; one recent report described angiographic reperfusion following local administration in CRAO (11), but systematic evaluation is still lacking.

Taken together, these clinical data delineate a pragmatic framework for the potential use of PGE₁ in acute ocular ischemic disease. When given intravenously and sufficiently early, PGE₁ can improve retinal or optic-nerve perfusion and may translate into meaningful visual recovery, with an excellent safety profile. These findings, while preliminary, justify further controlled, multicenter studies aimed at defining optimal dosing, duration, and therapeutic combinations, as well as establishing imaging biomarkers for patient selection.

## 6 Unmet needs and limitations of current evidence

Despite promising clinical signals, the evidence base for PGE₁ in ocular ischemic disorders remains preliminary and marked by substantial gaps. The most conspicuous limitation is the small sample size of virtually all published investigations. For example, the largest dedicated cohort to date included just 21 eyes treated with intravenous liposomal PGE₁ in acute CRAO (8) — far below the numbers required to robustly detect moderate treatment effects or stratify by sub-group. Even aggregated meta-analysis across six studies accounted for only 21 total patients in retinal artery/optic nerve ischemia (9). This under-powering increases the risk of false-positive findings, publication bias and limits the ability to assess rare adverse events or differential responses across demographic or clinical phenotypes.

An additional and clinically relevant limitation concerns systemic safety in real-world patient populations. Individuals affected by retinal ischemic events are frequently elderly and carry significant cardiovascular comorbidity. Although intravenous PGE₁ has been well tolerated in published ophthalmic series, with no major hypotensive or hemorrhagic events reported, the limited size of these cohorts precludes definitive safety conclusions. The known systemic vasodilatory and antiplatelet effects of PGE₁ underscore the need for cautious patient selection and strengthen the rationale for local, targeted, or sustained-release delivery strategies designed to minimize systemic exposure.

Another major hurdle arises from the heterogeneity of patient populations, disease entities and endpoints. The published studies mix central and branch retinal artery occlusion, non-arteritic optic neuropathy variants, and even diabetic microangiopathy, often with varied time-to-treatment intervals, baseline perfusion status and concomitant therapies. Endpoints likewise diverge — ranging from Snellen or logMAR VA changes, to OCT thickness, vessel diameter metrics, optical-intensity ratios, and perfusion changes on fluorescein angiography (8, 10) — making direct comparisons difficult and precluding meta-analytic pooling with confidence. Moreover, structural biomarkers (e.g., maximal retinal thickness, optical-intensity ratio) show promise but lack standardization or validation in this setting (10).

A further drawback is the uncertainty around optimal delivery and pharmacokinetics. Most clinical experience to date has involved systemic (intravenous) infusion of PGE₁ over 5–14 days, sometimes followed by oral continuation (8, 34). Yet human pharmacokinetic data reveal rapid plasma metabolism of PGE₁ which is rapidly metabolized into inactive compounds, such as 15-keto-13,14-dihydro-PGE₁ within minutes after infusion cessation (40, 47). The extent to which such systemic dosing achieves sufficient intra-ocular tissue concentrations over a meaningful duration — and whether local (intra-arterial or intra-ocular) delivery might improve target bioavailability — remains unknown. A rare case report of intra-arterial PGE₁ administration documented angiographic reperfusion (11), whereas earlier experiences with intravenous infusion also demonstrated partial restoration of retinal flow in acute CRAO (48), supporting the biological plausibility of vasodilator-mediated reperfusion though systematic data remain limited.

Long-term outcome data are notably sparse. Most published studies follow patients for 1 to 3 months post-treatment; beyond 6 months, data are largely anecdotal [for example, one diabetic microangiopathy case followed 4.5 years (45)]. Without long-term follow-up, the durability of functional and anatomic improvements remains uncertain, and the risk of late relapse or need for retreatment is unknown. Equally, late safety outcomes—especially in older patients with cardiovascular comorbidity—have not been fully explored.

**Figure 1:**
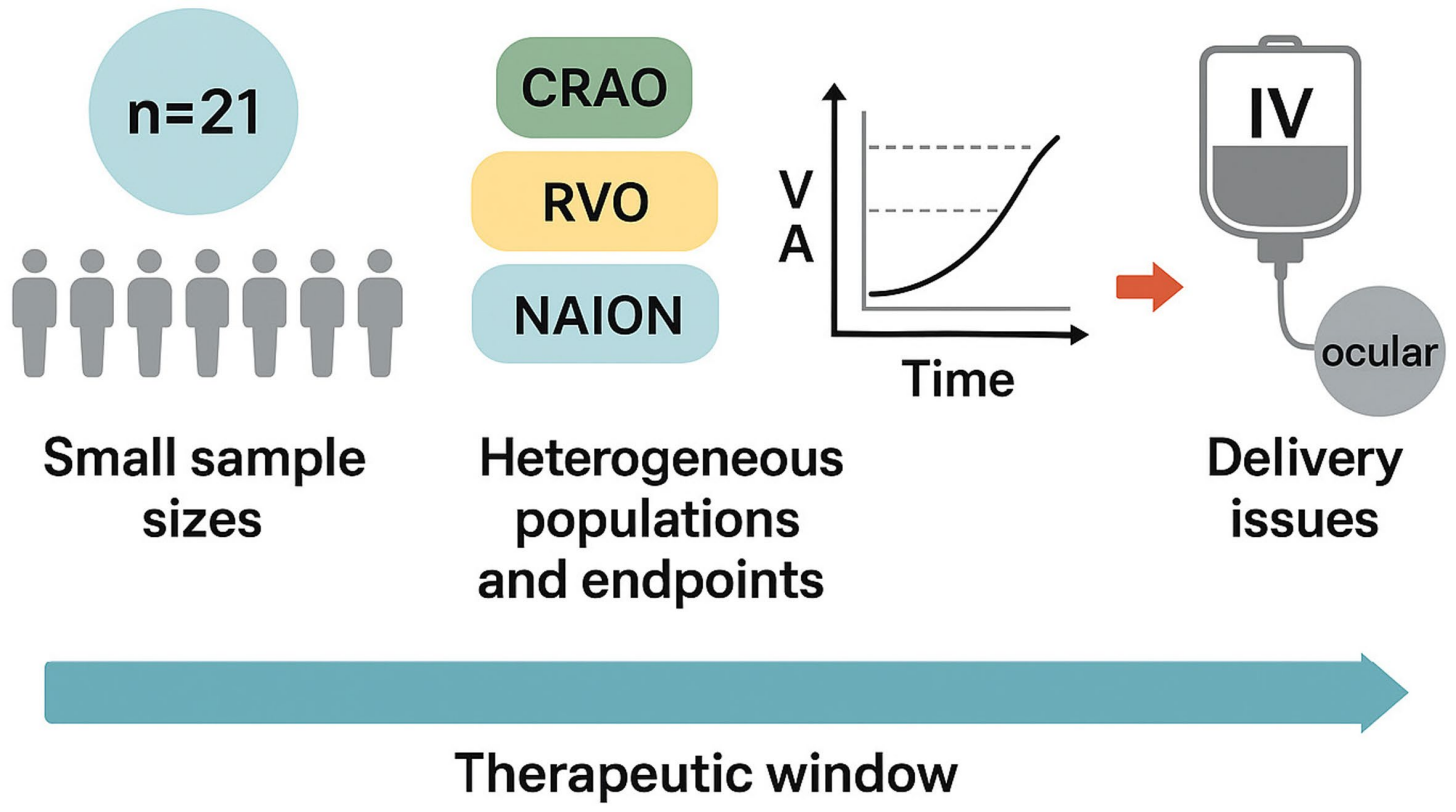
Unmet needs and evidence gaps in clinical research on Prostaglandin E₁ (PGE₁) for ocular ischemic disorders. The schematic summarizes the major limitations characterizing current clinical evidence on PGE₁ therapy. Most studies are limited by small sample sizes (typically ≤ 20 eyes), heterogeneous patient populations encompassing central retinal artery occlusion (CRAO), retinal vein occlusion (RVO), and non-arteritic ischemic optic neuropathy (NAION), and variable endpoints ranging from best-corrected visual acuity (VA) to angiographic or OCT-based perfusion indices. Additional gaps include uncertainty about drug delivery routes (intravenous vs. local ocular administration) and lack of long-term outcome data. The horizontal bar depicts the concept of a therapeutic window, emphasizing that treatment timing remains critical yet poorly standardized. Together, these factors underscore the need for well-powered randomized controlled trials with harmonized endpoints and optimized delivery strategies.

Finally, comparative or combinatorial therapy studies are absent. It remains unclear where PGE₁ fits into current clinical algorithms: for example, how it might complement or interact with intravitreal anti-VEGF therapy, corticosteroids, systemic reperfusion strategies or neuroprotective agents. In other ischemic vascular fields, meta-analysis suggests PGE₁ may reduce major adverse cardiac events in reperfusion settings (49), but analogous ocular data do not exist. Hence the lack of head-to-head comparisons or factorial trial designs limits our ability to define PGE₁‘s added value within an evidence-based therapeutic framework (Figure 1).

Taken together, these limitations indicate that PGE₁ cannot presently be recommended for routine clinical use in ischemic retinal diseases. Its current role should be confined to clinical trials or structured investigational protocols, ideally within academic or multicenter settings. Future progress will depend on randomized controlled studies with harmonized endpoints, standardized imaging metrics, and clearly defined inclusion criteria, particularly in acute CRAO.

In light of the limitations outlined above, the next chapter will explore emerging frontiers in the use of Prostaglandin E₁ for ocular ischemia — including novel delivery strategies, biomarker-guided patient selection, combinatorial therapies, and design considerations for prospective randomized trials.

## 7 Future perspectives

Future clinical development of PGE₁ in ophthalmology should proceed in a prioritized and indication-specific manner. Based on current mechanistic rationale and available human data, acute central retinal artery occlusion emerges as the most suitable primary target for prospective randomized trials. Other ischemic retinal disorders, including RVO and diabetic retinopathy, may be explored in later phases once disease-specific biomarkers, delivery strategies, and safety profiles are better defined.

### 7.1 Novel drug-delivery strategies

The short plasma half-life of PGE₁, along with its extensive first-pass pulmonary metabolism, poses a major barrier to durable intraocular exposure following systemic administration (40, 47). Recent advances in ocular pharmacology have opened the possibility of localized, sustained-release delivery systems designed to maintain therapeutic concentrations while minimizing systemic effects. Biodegradable intravitreal implants based on poly(lactic-co-glycolic acid) (PLGA) or lipid matrices can achieve controlled prostaglandin release for weeks to months, a technology already validated for corticosteroids and non-steroidal anti-inflammatory drugs (50). Experimental formulations of prostaglandin-mimetic or vasoprotective nanoparticles have demonstrated targeted endothelial uptake, improved retinal penetration, and enhanced neurovascular protection in rodent ischemia models (51). In particular, PGE₁-containing polymeric nanoparticles have been shown to restore perfusion and functional recovery in experimental vascular ischemia, confirming the feasibility of nanoparticle-based delivery for prostaglandin compounds (52). In parallel, hydrogel-based sustained-release systems are emerging as promising scaffolds for long-term intravitreal delivery (53). Similarly, microneedle-assisted transscleral delivery and subconjunctival depot systems are under investigation to achieve sustained periocular release with minimal invasiveness (54). However, a topical application of PGE₁ directly to the ocular surface would likely provoke undesirable effects, including vasodilation and conjunctival hyperemia (primarily via EP_4_ receptors) and inflammatory responses (via EP_1_/EP_3_ receptors). In contrast, transdermal or subcutaneous routes—already validated for other prostaglandins and for PGE₁ itself in the treatment of erectile dysfunction—could provide a safer and more practical alternative for systemic delivery (55). Transdermal patches and microneedle-based cutaneous systems have been successfully employed for controlled prostaglandin absorption in non-ocular indications, achieving steady plasma concentrations while minimizing peaks that cause hypotension or flushing (56, 57). These results, although extrapolated from systemic applications, support the feasibility of cutaneous delivery as an alternative to intravenous infusion. Such platforms could serve as non-invasive substitutes for intravenous infusion, provided that ocular pharmacokinetics and retinal tissue exposure are rigorously characterized. Future research should focus on optimizing cutaneous sustained-release formulations of PGE₁, which could overcome the pharmacokinetic limitations of systemic infusion and provide a practical, non-invasive approach for chronic neurovascular maintenance therapy.

### 7.2 Combination therapeutic approaches

Given the multifactorial nature of ischemic retinal injury—encompassing vascular constriction, oxidative stress, inflammation, and secondary neuronal degeneration—monotherapy is unlikely to yield maximal benefit. Synergistic combinations of PGE₁ with established pharmacological agents merit systematic evaluation. Anti-VEGF drugs, while highly effective in reducing macular edema (25), do not address the upstream vascular tone and perfusion deficits that PGE₁ may improve (34). Thus, a dual-approach combining PGE₁ with anti-VEGF therapy could theoretically restore perfusion while simultaneously limiting vascular leakage, a hypothesis supported by preclinical evidence showing that PGE₁ exerts endothelial-protective and anti-apoptotic effects under oxidative and hypoxic stress, promoting vascular stability and cell survival through modulation of HIF-1α signaling and attenuation of reactive oxygen–induced injury (58, 59).

The integration of PGE₁ with neuroprotective or anti-inflammatory agents—such as citicoline, brimonidine, or minocycline—also warrants exploration. PGE₁ has been shown to engage EP receptor signaling (notably EP4) and enhance cAMP–PKA activity, a pro-survival axis demonstrated in recent mechanistic work (60). In parallel, PGE₁ exerts endothelial-protective and anti-apoptotic effects in cell and preclinical models (58, 59). Several clinically relevant neuroprotective/anti-inflammatory agents (citicoline, brimonidine, minocycline) have independent evidence of protecting retinal ganglion cells and reducing inflammation/vascular permeability in optic-nerve and retinal ischemia models (61–63). Together, these data justify exploring combined PGE₁ and neuroprotective agent regimens. The cAMP-linked survival signaling downstream of EP₂/EP₄-receptor activation—recently demonstrated as a key neuroprotective mechanism in retinal ganglion cells (64) and reviewed as a major pro-survival pathway in ocular and neural tissues (65)—could plausibly synergize with the anti-apoptotic mechanisms of these agents. However, direct combination studies in ocular models are still needed. Beyond pharmacological synergy, the therapeutic benefit of PGE_1_-induced vasodilation can be complemented by multimodal strategies, such as remote ischemic conditioning (RIC) (66) or low-level light therapy (LLLT) (67), both of which are known to directly enhance mitochondrial resilience and function (68, 69).

### 7.3 Imaging biomarkers for patient selection and monitoring

The clinical success of PGE₁ therapy will ultimately depend on the ability to identify patients most likely to benefit and to monitor perfusion recovery objectively. Recent advances in optical coherence tomography angiography (OCTA) and wide-field perfusion imaging have revolutionized quantitative assessment of retinal microcirculation. Parameters such as vessel-density index, perfused capillary area, and flow-deficit metrics correlate closely with functional recovery in CRAO and RVO (70, 71) Baseline OCTA parameters could therefore serve as predictive biomarkers for responsiveness to vasodilatory therapies like PGE₁, while longitudinal imaging enables dynamic evaluation of treatment efficacy. The development of standardized imaging endpoints will also facilitate inter-study comparability and could bridge mechanistic and clinical research domains.

Emerging modalities such as functional retinal oximetry and laser speckle flowgraphy are likewise expanding insight into microvascular dynamics and oxygen delivery (72, 73). Integration of these quantitative imaging biomarkers into future trials would enable early detection of perfusion changes preceding visual improvement, thereby refining therapeutic windows and patient-selection criteria.

### 7.4 Translational research and neurovascular integration

Beyond its hemodynamic effects, PGE₁ exerts downstream influence on mitochondrial integrity, endothelial barrier stability, and neuronal survival. Activation of EP₂/EP₄ receptors engages G_s-mediated cAMP–PKA signaling (74), and cAMP–PKA activity has been shown to preserve mitochondrial supercomplex organization and respiratory efficiency (75); moreover, PGE₁ protects endothelial cells from oxidative injury (58), while EP₂-agonism confers cAMP-dependent neuroprotection in retinal ganglion cells (64), together supporting the proposition that PGE₁ can influence mitochondrial integrity, endothelial barrier stability and neuronal survival via EP-linked cAMP–PKA pathways. This positions PGE₁ as a unique pharmacological bridge between vascular and neuronal protection, aligning with contemporary models of the retina as a neurovascular unit. Translational research should therefore focus on defining molecular markers of PGE₁-induced neurovascular coupling, possibly through combined single-cell transcriptomic and metabolomic profiling in experimental ischemia, to identify cell-type-specific molecular targets and signaling pathways responsive to PGE₁ (76). Such integrative studies may reveal cross-talk with established neurotrophic pathways, including BDNF and NGF signaling, thereby guiding the rational design of next-generation analogues with improved receptor selectivity or resistance to enzymatic degradation. The long-term vision is to evolve PGE₁ therapy from an emergency vasodilator into a chronic neurovascular modulator, administered through sustained-release technologies and guided by precision imaging and molecular diagnostics.

## 8 Conclusion

Prostaglandin E₁ (PGE₁) has emerged as a therapeutic candidate with the rare ability to target both the vascular and neuronal consequences of retinal ischemia. Its integrated action on microcirculatory function and cell survival provides a coherent rationale for further clinical development.

Despite encouraging early signals—particularly in early-treated CRAO—the current clinical evidence is insufficient to support routine use of PGE₁ in ischemic retinal diseases. At present, PGE₁ should be regarded as an investigational or adjunctive therapy, suitable for evaluation within controlled clinical trials rather than standard practice. Clear acknowledgment of these limitations is essential to avoid premature clinical translation.

The pharmacological challenges of PGE₁ -its short systemic half-life and metabolic instability- have historically limited its broader development. Innovative sustained-release systems, including liposomal, transdermal, and microneedle-based platforms, now provide realistic opportunities to overcome these barriers, enabling chronic administration with minimal invasiveness. Parallel advances in high-resolution vascular imaging and molecular profiling promise to refine patient selection and response monitoring, thereby enhancing the translational potential of PGE₁ in ocular ischemia.

It is worth reflecting that the most convincing clinical evidence for PGE₁ in retinal ischemia dates back more than three decades, yet no approved formulation has reached the market. The reasons for this translational gap likely extend beyond scientific uncertainty, encompassing economic, regulatory, and patent-related obstacles that have deterred sustained pharmaceutical investment in an off-patent molecule.

**Figure 2:**
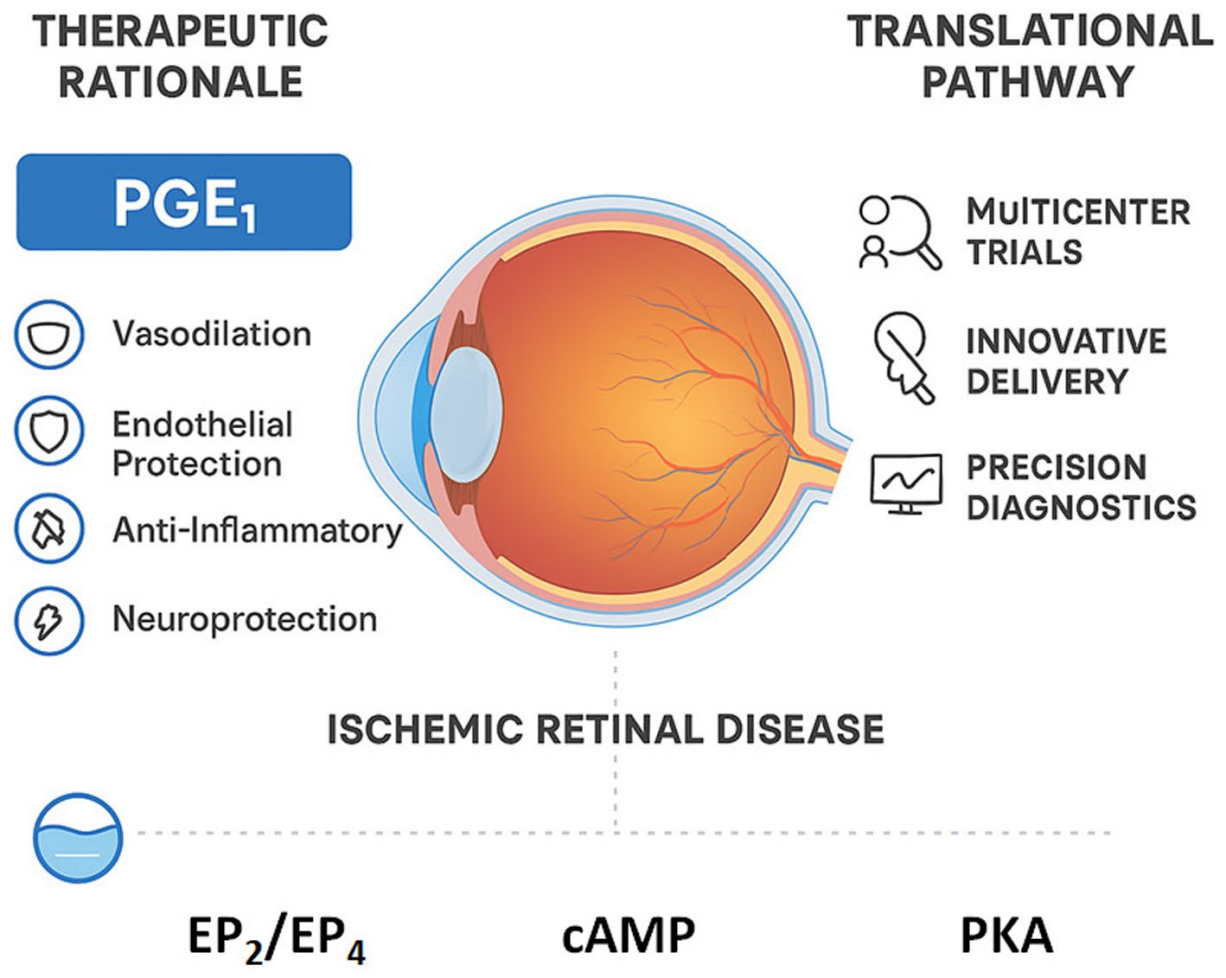
Therapeutic rationale and translational pathway of prostaglandin E_1_ (PGE_1_) in ischemic retinal disease. PGE_1_ activates the EP_2_/EP_4_–cAMP–PKA pathway, promoting vasodilation, endothelial protection, and neuroprotection to restore retinal perfusion and neuronal survival. The diagram integrates these mechanisms with emerging delivery strategies (liposomal, transdermal, microneedle) and highlights the need for multicenter trials and precision diagnostics to translate PGE_1_ from off-label use to an established therapy for retinal ischemia.

Nonetheless, the pathophysiological rationale remains strong, and the convergence of modern drug-delivery and precision-diagnostic technologies may finally allow PGE₁ to realize its therapeutic potential. It is our hope that this review will stimulate renewed scientific and clinical interest, encouraging a new generation of studies that could ultimately establish PGE₁ as an official, evidence-based treatment for ischemic retinal diseases, where it currently remains an off-label but promising therapeutic option (Figure 2).
